# Challenges related to records and quality of information in the Amazon

**DOI:** 10.1590/0102-311XEN098323

**Published:** 2023-08-11

**Authors:** Bernardo Lanza Queiroz

**Affiliations:** 1 Departamento de Demografia, Universidade Federal de Minas Gerais, Belo Horizonte, Brasil.

One of the United Nations’ sustainable development goals is the establishment of high-quality, valid, and reliable civil registry and vital statistics systems necessary for the design, evaluation, and implementation of social, economic and health programs, especially in the context of changes in the pattern of mortality that many countries have been experiencing. Although several methods developed in demography ^1^ ^,^ ^2^ allow for following the evolution of the quality of the records and to indirectly estimate the levels of mortality in different localities, the quality of the system of vital statistics allows tracking the development of health conditions in a population, evaluating the public health policies, and assisting the health managers ^3^ ^,^ ^4^ ^,^ ^5^.

Recently, the quality of mortality data in Brazil has improved substantially. Estimates of the degree of coverage of death registries in Brazil increased from about 80% in the 1980s to about 95% in 2010 ^6^ ^,^ ^7^ ^,^ ^8^. However, regional variability in the quality of the degree of coverage of the death registry and the quality of information on the causes of death in the country is still high ^6^ ^,^ ^7^ ^,^ ^9^. Since 2010, the South and Southeast regions show a complete coverage of adult mortality records. The states of the Northeast and North, even with the trends of improvements in recent years, have locations with low coverage ^6^ ^,^ ^10^. The average coverage of mortality in the North Region rose from 65% to 76% from 1980 to 2010, whereas coverage in the South Region increased from 95% to 98% in the same period ^10^. In the case of smaller areas, such as mesoregions, the impacts of data quality can be even greater. The estimated degree of coverage for the São Paulo metropolitan area is 100%, high quality of data, with the probability of adult death of 0.2364, for individuals aged from 15 to 60 years. In the case of the South Amazonian mesoregion, the estimated degree of coverage is 68%. The observed data indicate a probability of adult death of 0.1102; however after applying the correction it is estimated at 0.1621 ^10^. An exercise with data from the *2010 Demographic Census* indicates, for infant mortality, coverage of the events registration of practically 100% in the São Paulo metropolitan area compared with about 50% in the mesoregion of the South Amazon. In summary, directly analyzing the data, without applying correction methods, can lead to erroneous results directly affecting health policies. An important conclusion, however, is that the different estimation methods applied to limited data may present quite different results, complexifying the definition of health strategies. A study comparing different estimates of mortality for Brazil and its regions shows the great discrepancy of these estimates and highlights the importance of continuous investment in the quality of records and in the replicability of the methods used ^11^.

In the specific case of the Amazon Region, the under-registration of deaths is high ^9^ ^,^ ^10^. The comparative study of the registration of deaths from different sources (Demographic Census, Civil Registry, and Mortality Information System - SIM) for 2010 ^12^ shows that in almost 65% of the municipalities of the North Region, the enumeration of deaths in the *2010 Demographic Census* is higher than the registration of deaths in the SIM and in the Civil Registry. The study also shows that the census data have a better enumeration of events in locations with a higher rate of deprivation and worse social and economic conditions in the North Region. The release of the *2022 Demographic Census* data will allow for similar analyses and assessments for the most recent period.

The improvement in the recording of events and the regional differentials tell only part of the story. In addition to enumerating the events, collecting additional information, especially the underlying causes of death is essential ^12^ ^,^ ^13^ ^,^ ^14^. Over the last three decades, the percentage of deaths recorded as ill-defined causes in Brazil has decreased from 27.5% in 1980 to 8.5% in 2010. In the states of the Amazon, in the same period, about 13% of deaths are registered as ill-defined causes ^10^. In 2019, those were still 10% compared with less than 4% in the South Region.

Brazil is marked by great regional differences in the levels and trends of causes of death, reflecting different stages of the epidemiological transition process ^3^ ^,^ ^15^ ^,^ ^16^. Considering the monitoring of the objectives of sustainable development ^17^, especially maternal and child health and the reduction of maternal mortality, regional variability is high ^18^. For example, in 2016, maternal mortality reached 64.4 per 100,000 live births in Brazil, ranging from 44.2 in the South to 84.5 per 100,000 live births in the North Region. In the state of Amapá, the rate reaches 141.7 per 100,000 live births. One of the great challenges of improving actions aimed at reducing maternal mortality is the lack of quality estimates that have regional details and that consider the temporal trend. The differential in the quality of the record of the specific cause of maternal death hinders the correct measurement of the level and trends ^19^ ^,^ ^20^ ^,^ ^21^. The various methods applied based on Brazilian data may present very different results that have implications for the definitions of policies aimed at maternal and child health. Estimates based entirely on statistical models may present even more distinct results and in some cases have very varied trends among the studies ^22^ ^,^ ^23^. Another example of the importance of the quality of the registry of causes of death in the Amazon Region refers to the monitoring and measurement of the impact of neglected tropical diseases, with a significant weight on mortality and years of life lost ^24^ ^,^ ^25^.

Regarding more specific population groups, the limitations of the quality of vital statistics are particularly important for the adequate monitoring of the health conditions and mortality of indigenous populations. Since information on this group is very limited, most recent studies end up being based on information on deaths in households collected by the *2010 Demographic Census* ^26^ ^,^ ^27^ ^,^ ^28^. Although the censuses offer an opportunity to study the original populations in the Amazon Region, the temporal frequency limits the adequate monitoring of the health condition of this population, further reinforcing the need to invest in the quality of the system of recording vital statistics for the different population sub-groups in Brazil.

Despite the improvement in the quality of death records in recent decades, the regional differential is still large and the limitations in the registration of underlying cause, age declaration, and various socioeconomic information are significant. The various methodological advances in the estimates presented by researchers and agencies, despite their importance, have limitations and do not replace quality information systems. In short, all efforts should be directed toward producing high-quality records, including the cause of death. Advances in registries can produce better and more reliable mortality estimates for understanding trends and differentials for several developing countries ^5^ ^,^ ^29^. And, especially in the Amazon Region, these advances are indispensable for knowing the real panorama of health of the populations.
